# High Nitrogen Levels Alleviate Yield Loss of Super Hybrid Rice Caused by High Temperatures During the Flowering Stage

**DOI:** 10.3389/fpls.2019.00357

**Published:** 2019-03-26

**Authors:** Ke Liu, Jun Deng, Jian Lu, Xiaoyan Wang, Bilin Lu, Xiaohai Tian, Yunbo Zhang

**Affiliations:** ^1^Hubei Collaborative Innovation Center for Grain Industry, Yangtze University, Jingzhou, China; ^2^College of Agriculture, Yangtze University, Jingzhou, China

**Keywords:** super hybrid rice, nitrogen, high temperature, grain yield, heat stress, climate change

## Abstract

The effect of high temperatures on rice production has attracted considerable research attention. It is not clear, however, whether nitrogen (N) management can be used to alleviate the damaging effects of high temperatures on flowering in rice. In this study, we compared the yields of five elite super hybrid rice varieties and examined their heat tolerance under four N treatments in two seasons with contrasting temperatures at flowering: 2015 (normal temperature) and 2016 (high temperature). The average daily temperature during the flowering stage in 2016 was 31.1°C, which was 4.5°C higher than that in 2015. There was a significant positive correlation between grain yield and N level (*R*^2^ = 0.42, *P* < 0.01). However, mean grain yield of the five rice varieties in 2015 was 10.5% higher than that in 2016. High N levels reduced yield losses in plants exposed to high temperature in 2016. The mean seed-set percentage in 2016 was 13.0% lower than that in 2015 at higher N levels, but spikelets per panicle increased by 7.6% at higher N levels compared with lower N levels. Higher N levels reduced the number of degenerated spikelets under high temperatures. Spikelets per panicle and N treatment level were positively correlated at high temperatures (*R*^2^ = 0.32, *P* < 0.05). These results confirmed that increasing N application could alleviate yield losses caused by high temperatures in super hybrid rice during the flowering stage.

## Introduction

Mean global temperatures are forecast to rise by as much as 2°C by the middle of the 21st century, with a concurrent increase in the frequency and intensity of extreme heat waves (Intergovernmental Panel on Climate Change [IPCC], 2013). Piao et al. (2010) reported that temperatures have increased by 0.15–0.40°C every 10 years over the past 50 years in China. As the largest rice producer in the world, China contributes nearly thirty percent of global rice production (Food and Agriculture Organization [FAO], 2010; Seck et al., 2012). Rice cultivars in China are increasingly exposed to extremely high temperatures with ongoing climate change. For example, temperatures of 35°C–significantly exceeding the critical threshold temperature (33°C) for growing rice (Bheemanahalli et al., 2016; Liu et al., 2017)–that lasted for more than half a month, contributed to an estimated total yield loss of 5.18 × 10^7^ t in the Yangtze Valley, resulting in significant economic losses in 2003 (Tian et al., 2009). The annual planting area of hybrid rice has increased to about 15 million ha in recent years, accounting for more than 50% of the total rice planting area in China (Cheng et al., 2007). The super hybrid rice cultivars that have made significant contributions to the increase in yields in China (Peng et al., 2008) are predominant in the Yangtze Valley. It is crucial, therefore, to alleviate the effects of heat stress on rice production in this area to maintain food security in China.

Flowering is considered the most susceptible stage of development to high-temperature stress in rice plants (Satake and Yoshida, 1978; Farrell et al., 2006). Temperatures over 35°C for more than 1 h during flowering can lead to significant declines in spikelet fertility; a negative correlation between spikelet fertility and cumulative temperature over 34°C in rice plants was observed by Jagadish et al. (2007). Shorter grain filling durations caused by high temperatures were due to the loss of sink activity owing to earlier senescence of the panicle (Kim et al., 2011). Previous studies have indicated that an increase in spikelet sterility caused by high-temperature stress was caused by abnormal anther dehiscence (Matsui and Omasa, 2002), glume closure (Yan et al., 2017) and impaired pollination (Matsui et al., 2005) and pollen germination (Jagadish et al., 2009). In the Yangtze Valley, Tian et al. (2009) observed that a maximum temperature of approximately 35°C or daily mean temperature of 30°C (with relative humidity of approximately 70% and low wind speeds) lasting for more than 3 days during the flowering period, led to significant seed-set losses in rice plants.

Some previous studies have shown that plant nutrients play a vital role in improving tolerance to temperature stress and that proper nitrogen management can partially mitigate the damaging effects of high temperature on crops (Waraich et al., 2012). A high N rate at panicle initiation or flowering was shown to alleviate the detrimental effects of high temperature on grain yield (Dai et al., 2009; Duan et al., 2013; Yang et al., 2014). One possible reason for this is that N application reduced the rice canopy temperature by contributing to a better rice canopy structure with a high leaf area index (LAI), which facilitated higher transpiration cooling (Yan et al., 2008). However, Shi et al. (2017) reported that increasing total N fertilizer application did not alleviate the adverse effects of high night temperatures on rice yield. These results may be controversial in some cases as most results were obtained from climate chambers instead of field conditions. Little information is available about the effects of N in combination with high-temperature exposure on rice yields under field conditions. In this study, field experiments were carried out with the following objectives: (1) verify the beneficial effects of high N levels on grain yield under high temperatures and paddy conditions; (2) compare the yields of five elite super hybrid rice varieties under four N treatments; and (3) identify the key factors that influence grain yield of rice under high N conditions, when plants are subjected to high temperatures at the flowering stage.

## Materials and Methods

The study consisted of two experiments. Pot experiments were used to investigate the heat tolerance of different rice varietals, and field experiments were used to verify the effects of high N on grain yields of super hybrid rice cultivars under conditions of high temperature during the flowering period.

### Pot Experiments

Five super hybrid rice cultivars (Table 1), were used in this study. Seeds of these cultivars were germinated at room temperature (25°C) during the second half of April. After 20 days, 25–30 rice seedlings of similar height were transferred into plastic pots (30 cm height and 30 cm diameter). For each pot, 12.5 kg of soil was mixed with 8 g of composed fertilizer (with a ratio of 26:10:15 N:P:K) and then filled with water. Only the main stem was kept. Pots were then cultivated at the experimental farm of Yangtze University, Jingzhou City, Hubei Province, China (112°31^′^E, 30°21^′^N).

**Table 1:** Information about rice varieties used in the experiment.

Variety	Type	Year of release	Female parent	Male parent
LYPJ	Intermediate^a^	1999	Pei’ai64S	Yangdao 6
YLY1	Indica	2006	Y58S	Yangdao 6
YLY 2	Indica	2006	Y58S	Yuanhui 2
YLY 900	Indica	2015	Y58S	R900
S1000	Indica	Unreleased	Guangxiang24S	R900


*^*a*^The intermediate type between indica and japonica.*

At the anthesis stage, rice plants were exposed to high-temperature treatments for 3 days, as follows. 1 day before anthesis, the rice plants were moved into a growth chamber (Conviron Company, PGW40, Winnipeg, MB, Canada) to start heat treatment with a 14:10 h day:night cycle and a 2 h change in the temperature simulating typical local heat stress, following the protocol of Mu et al. (2017). For the control and high-temperature treatments, temperatures were set as shown in Table 2; the actual temperature regimes were 27°C (normal), and 30°C (high) average temperature, and their corresponding daily maximum temperatures were 31 and 33°C. The relative humidities were 70 and 80% for day and night, respectively.

**Table 2:** Day/night temperature settings (°C) of growth chambers for different heat treatments.

Treatment	Time (hh:mm)																							
	21:00	22:00	23:00	0:00	1:00	2:00	3:00	4:00	5:59	6:00	7:00	8:00	9:00	10:00	11:00	12:00	13:00	14:00	15:00	16:00	17:00	18:00	19:00	20:01
Normal	28	28	27	26	26	25	25	25	25	25	26	26	27	28	29	30	30	31	31	30	30	29	29	28
temperature
High	30	30	29	29	28	28	27	27	27	27	28	28	30	30	32	32	33	33	32	32	31	31	30	30
temperature


To examine the seed-set percentage, panicles were sampled 1 month after heat-stress treatment. Twenty panicles were sampled from each pot and their seed sets were examined by manual inspection of ovary development.

### Field Experiments

Field experiments were conducted at the experimental farm of Yangtze University in 2015 and 2016. Soil samples from the upper 20 cm were taken before the experiments and soil properties were tested following the protocol of Lu (1999). The soil of the experimental site was calcareous alluvial with the following properties: pH 6.8, 18.5 g kg^-1^ organic matter, 110.5 mg kg^-1^ alkali-hydrolysable N, 25.0 mg kg^-1^ available P, and 105.5 mg kg^-1^ available K. Data for the soil properties were averaged across the 2 years.

Treatments were arranged in a split-plot design with N treatments as the main plots and cultivars as the subplots. The experiment was replicated three times, and the subplot size was 30 m^2^. The four N treatments were applied as follows: 0 kg ha^-1^ (N1), 210 kg ha^-1^ (N2), 300 kg ha^-1^ (N3), and 390 kg ha^-1^ (N4). Nitrogen fertilizer was applied at the basal, tillering, and panicle stages in a ratio of 5:2:3. For the N2 treatment, 105, 42, and 63 kg N ha^-1^ were applied at the baseline (1 day before transplanting), early tillering (7 days after transplanting), and panicle initiation stages (the first appearance of a differentiated apex), respectively. For the N3 and N4 treatments, 150, 60, and 90 kg N ha^-1^ and 195, 78, and 117 kg N ha^-1^ were applied at the baseline, early tillering, and panicle initiation stages, respectively.

Five super hybrid rice cultivars were used in the experiments (Table 1). Pre-germinated seeds were sown in a seedbed at a rate of 25 g m^-2^. Thirty to 32-day-old seedlings were transplanted, with a hill spacing of 20 × 30 cm and two seedlings per hill. Phosphorus (105 kg ha^-1^) was applied and incorporated in all subplots 1 days before transplanting. Potassium (210, 300, and 390 kg ha^-1^) was split equally between the basal and panicle initiation stages under N2, N3, and N4, respectively. Crop management followed standard cultural practices. Insects were intensively controlled by chemicals to avoid biomass and yield losses.

Twelve hills were sampled diagonally from a 5 m^2^ harvest area in each subplot at maturity. For all sampling, the three border lines were excluded to avoid border effects. Plants were hand threshed after the panicles were counted. Filled spikelets were separated from unfilled spikelets by submerging them in tap water. Three subsamples of 30 g filled grains and all unfilled spikelets were removed to count the number of spikelets. The filled and unfilled spikelets were determined after oven drying at 70°C to a constant weight. Spikelets per panicle and seed-set percentage (100 × filled spikelet number/total spikelet number) were calculated. Grain yield was determined from a 5 m^2^ area in the middle of each subplot and adjusted to a moisture content of 0.14 g H_2_O g^-1^ fresh weigh.

To address the yearly experimental variation and measure the changes in yield production caused by N, ΔG was used to represent the grain yield production increase from using N, calculated as follows:

$$\Delta {\text{G}}_{\text{N}}{}_{i}=\text{Mean grain yield in N}i-\text{mean grain yield in N}1,$$

where *i* is treatment 2, 3, or 4.

To test the effects of cultivars, treatments, year, and their interaction effects on grain yield and yield components, statistical analysis was carried out using a three-way analysis of variance (ANOVA) using SAS software (SAS Institute Inc., Cary, NC, United States); a least significant difference (LSD) test was used to compare the means.

## Results

### Heat Tolerance of the Tested Cultivars

**FIGURE 1:**
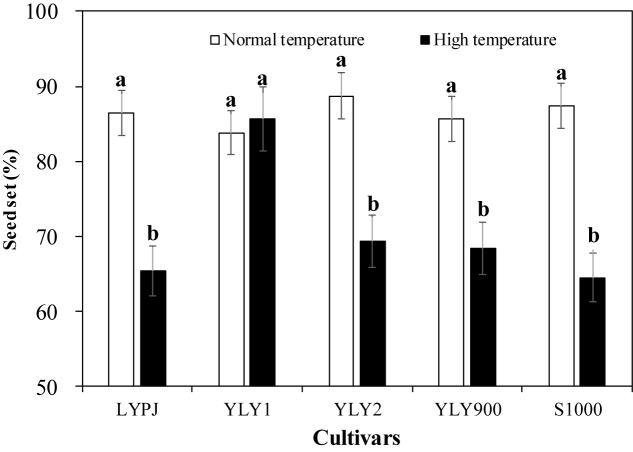
Heat tolerance of tested cultivars under growth chamber conditions. Different lowercase letters indicate significant differences at *P* < 0.05 between the control (normal temperature) and heat treatment (high temperature).

The cultivars responded differently to high-temperature treatment. Seed-set percentages for all cultivars, except YLY1, were significantly reduced by high temperature (Figure 1). There was a 24.4% difference in seed set in LYPJ between normal and high temperature, 21.9% in YLY2, 20.2% in YLY900, and 26.2% in S1000. YLY1 did not show any significant change in seed set under high temperature, which suggests that YLY1 was the most tolerant to heat stress among these cultivars.

### Temperature Differences in 2015 and 2016 During the Flowering Period

**FIGURE 2:**
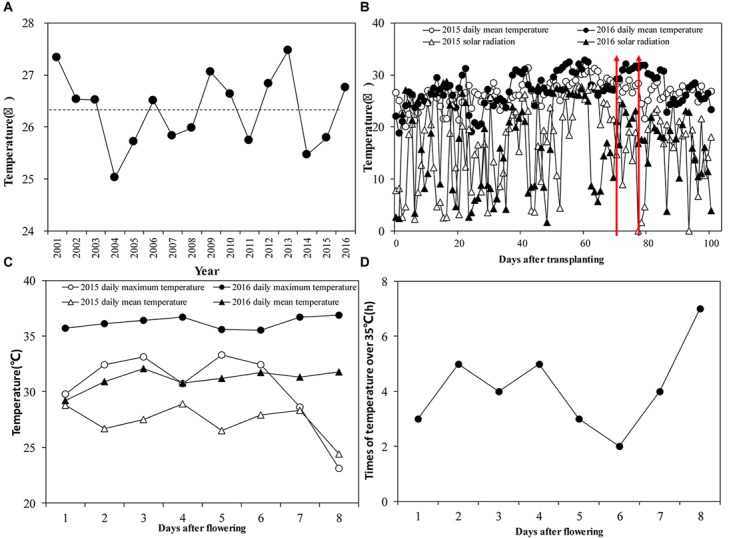
**(A)** Seasonal mean temperature from June to September of 2001–2016. The dotted line is the average seasonal mean temperature; **(B)** Daily mean temperature and solar radiation from transplanting to maturity in 2015 and 2016. The red arrows indicate the flowering stage at Jingzhou in 2015 and 2016; **(C)** Average daily mean temperature during the flowering stage in 2015 and 2016; **(D)** Duration of temperatures >35°C during the flowering stage in 2016.

For the period 2001–2016, the summer mean temperature was 26.3°C (Figure 2A). Among these years, only four had temperatures below the historical seasonal mean, which were 2004, 2011, 2014, and 2015. In 2015, the seasonal mean temperature was only 25.7°C but in some years it was extremely high. The fourth highest seasonal mean temperature was 26.8°C in 2016. Daily average temperatures during the growing season in 2016 were 1.0°C higher than those in 2015 (Figure 2B). The daily mean temperature during the flowering period in 2016 was 31.2°C, compared to 26.7°C in 2015. There was a 3.4% difference in average daily solar radiation between the 2 years during the growing season: seasonal average daily radiation was 15.7 MJ m^-2^ d^-1^ in 2015 and 16.1 MJ m^-2^ d^-1^ in 2016. During the flowering stage, the daily highest temperature in 2016 was 36.9°C, while it was only 33.3°C in 2015 (Figure 2C). There was a significant difference between the 2 years in the duration of temperatures exceeding 35°C during the flowering period. In 2015 there were no days with temperatures over 35°C during the flowering period. In 2016, however, temperatures over 35°C were observed throughout the flowering period, lasting from 2 to 7 h d^-1^ (Figure 2D).

### Grain Yield, Seed-Set Percentage, and Spikelets per Panicle

The ANOVA results for grain yield, seed-set percentage, and spikelets per panicle are shown in Table 3. Planting year (Y) had a significant effect on all parameters. Grain yields differed significantly (*P* < 0.01) among varietals (V) and N treatments (T), but there were no significant differences in spikelets per panicle or grain filling between cultivars. The interaction effect of Y × V was significant (*P* < 0.01) for all parameters. Significant interaction effects of Y × T on grain yield and seed-set percentage were observed, and grain yield and spikelets per panicle were significantly (*P* < 0.01) affected by the interaction of V × T. Interaction effects of Y × V × T were only significant for spikelets per panicle.

**Table 3:** Analysis of variance showing the effects of year, varietal, and N treatment on grain yield, grain filling, and spikelet per panicle.

Source of Variance	Grain yield	Grain filling	Spikelet per panicle
Year (Y)	^∗^	^∗∗^	^∗∗^
Varietal (V)	^∗∗^	ns	Ns
Treatment (T)	^∗∗^	ns	^∗∗^
Y^∗^V	^∗∗^	^∗∗^	^∗∗^
Y^∗^T	^∗∗^	^∗^	Ns
V^∗^T	^∗∗^	ns	^∗∗^
Y^∗^V^∗^T	ns	ns	^∗∗^


*Ns, non-significant; ^∗^significant at *P* < 0.05, ^∗∗^significant at *P* < 0.01.*

### Grain Yield Under Nitrogen Treatments in 2015 and 2016

**FIGURE 3:**
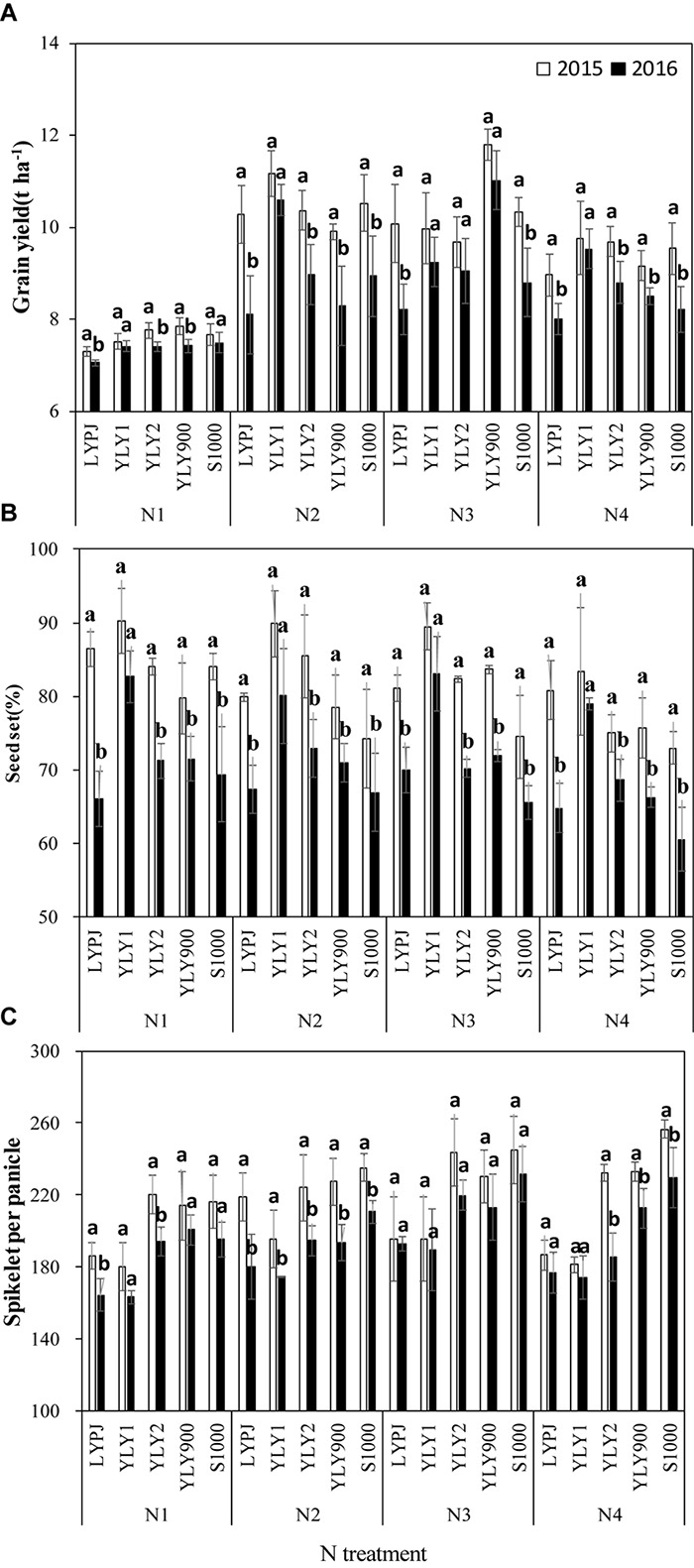
Grain yield **(A)**, grain filling **(B)**, and spikelets per panicle **(C)** of five super hybrid rice cultivars grown under different N treatments in Jingzhou, Hubei Province, China, in 2015 and 2016. Data are the means of three replicates and the vertical bars represent the standard error. Different lowercase letters indicate significant differences (*P* < 0.05). The four N treatments were 0 kg ha^-1^ (N1), 210 kg ha^-1^ (N2), 300 kg ha^-1^ (N3), and 390 kg ha^-1^ (N4).

Due to the negative effects of high temperature during the flowering stage in 2016, grain yields of all cultivars except YLY1 were significantly lower than those in 2015 under N1, N2, and N4 treatments (Figure 3A). For N3, some cultivars showed different response patterns to high temperature, and yield differences between years were not significant in YLY1, YLY2, and YLY900. Compared with the grain yields in 2015, grain yields of LYPJ and S1000 decreased by 18.6 and 14.8%, respectively, in 2016, while the range of annual yield differences in YLY1, YLY2, and YLY900 was only 6.5–7.3%.

**FIGURE 4:**
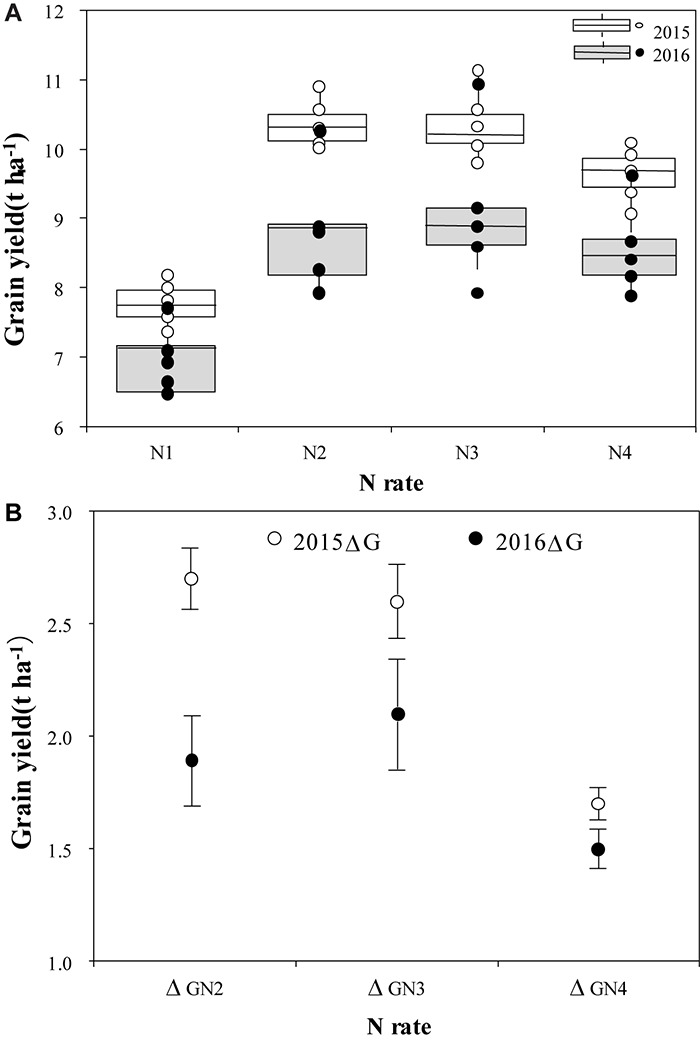
Grain yield of five super hybrid rice cultivars under different N treatments in 2015 and 2016 **(A)**, and average grain yields with grain yield production increase from using N (ΔG) **(B)**. The four N treatments were 0 kg ha^-1^ (N1), 210 kg ha^-1^ (N2), 300 kg ha^-1^ (N3), and 390 kg ha^-1^ (N4).

There were significant differences in grain yield between N treatments in 2015 and 2016 (Figure 4A). In 2015, the highest mean grain yield was 10.5 t ha^-1^ in the N2 treatment. As the N rates increased to 360 kg ha^-1^ (N4), mean grain yield decreased to 9.4 t ha^-1^. Compared to grain yield in 2015, mean grain yields were much lower in 2016 for all N treatments. The mean grain yield was highest (9.3 t ha^-1^) in N3, followed by 8.9 t ha^-1^ in the N2 treatment. In 2015, the differences in grain yield among cultivars within N treatments were not significant, however, there were significant differences in 2016 when plants were exposed to high temperature stress during the flowering stage; the grain yield of YLY1 was significantly higher than that of the other four cultivars. The difference between years in the production increase with N treatment was also significant (*P* < 0.05) (Figure 4B). The gap in ΔG between the 2 years decreased with an increase in N application rate; the yield difference in ΔG_N2_ was the highest (0.8 kg ha^-1^), and it was only 0.5 kg ha^-1^ in ΔG_N4_ and 0.2 kg ha^-1^ in ΔG_N3_.

### Seed-Set Percentage and Spikelets per Panicle Under Nitrogen Treatments in 2015 and 2016

High temperatures during the flowering stage significantly reduced the seed-set percentage of these cultivars (Figure 3B). Seed-set percentage in these cultivars, except in YLY1, was significantly lower in 2016 than in 2015. Increasing N resulted in a decrease in seed-set percentage. LYPJ was the most sensitive to heat stress, and its seed-set percentage decreased much more than that of the other cultivars with N treatments. The number of spikelets per panicle was significantly affected by the high temperature in 2016 (Figure 3C). Spikelets per panicle showed different responses to high temperature among N treatments. The differences in spikelets per panicle between years under N1 and N2 were much higher than those under N3 and N4. Compared with 2015, the spikelets per panicle decreased by 9.8 and 13.4% under N1 and N2, respectively, while it was only 5.4 and 7.6% under N3 and N4, respectively. The number of spikelets per panicle saw a significant increase under N3 in some cultivars, and there was no significant difference in spikelets per panicle between the 2 years.

### Correlations Between Grain Yield, Seed-Set Percentage, Spikelets per Panicle, and Nitrogen Treatment

**FIGURE 5:**
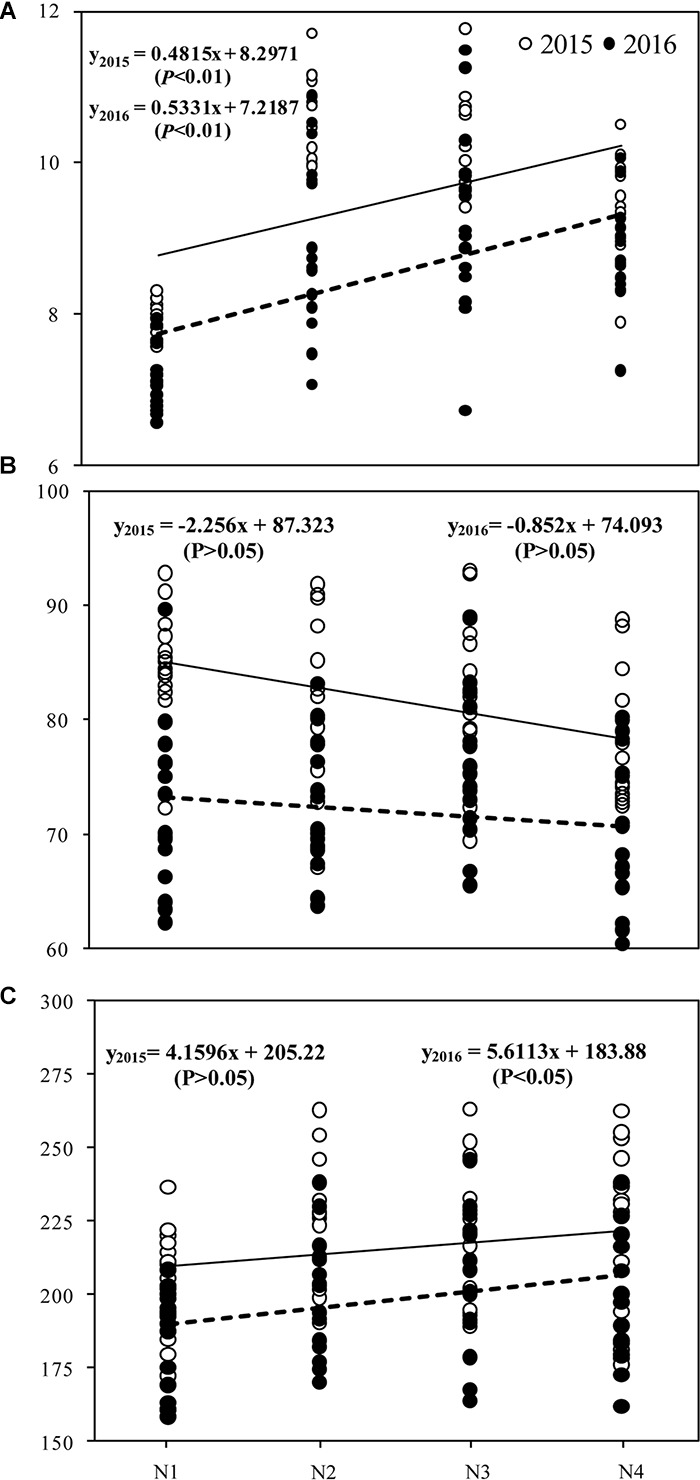
Correlation between grain yield **(A)**, grain filling **(B)**, spikelet per panicle **(C)**, and N treatments in 2015 and 2016. The four N treatments were 0 kg ha^-1^ (N1), 210 kg ha^-1^ (N2), 300 kg ha^-1^ (N3), and 390 kg ha^-1^ (N4).

There was a significant (*P* < 0.01) positive correlation between grain yield and N treatment, but no significant relationship was observed between seed-set percentage and N treatment (Figure 5). Grain yield (*P* < 0.01) and spikelets per panicle (*P* < 0.05) were positively correlated with N treatment in 2016.

## Discussion

Previous studies have reported the effects of high N levels on grain yields, under high-temperature stress (Duan et al., 2013; Yang et al., 2014). However, the results of these studies were obtained from climate chamber experiments, rather than field conditions. The responses of plants grown in the field can be very different from those in climate chambers (Chen et al., 2003). Therefore, these previous findings have limitations.

In our study, we compared the yields of super rice cultivars under different N treatments between 2 years in a field experiment. Because of a reduction in seed-set percentage caused by high temperature during the flowering stage, mean grain yield in 2016 was significantly lower than that in 2015. Despite the negative effect of high temperature, a lower proportion of yield loss was observed in those cultivars treated with higher levels of N (N3 or N4) than those in N1 or N2 treatments (Figure 2A); there were minor differences between ΔG_N3_ and ΔG_N4_. Our field results suggest that high N levels can positively affect grain yield when rice plants are exposed to high temperatures at the flowering stage.

However, Shi et al. (2017) argued that increasing total N did not alleviate the effects of high night temperatures on rice yields. This result may be due to the different varietals used. Heat-tolerant cultivars like YLY1 did not show significant differences between N treatments when exposed to high temperature, which suggests that some cultivars may not benefit from high N application to address yield loss caused by high-temperature stress. For other heat-sensitive cultivars, yield loss could be reduced by increasing N application rates. The yield performance of these heat-sensitive cultivars showed similar trends under high N levels when they were exposed to high-temperature stress in our study. Compared with the inbred cultivars used in Shi et al.’s (2017) experiment, the super hybrid rice cultivars in our study had larger panicles with numerous spikelets per panicle and high biomass production, and hence, required a large amount of N fertilizer input to achieve high yields. Varietal differences may, therefore, be responsible for the differences in results between studies.

The most recently released super hybrid rice cultivars have a large number of spikelets on a panicle, with a large yield potential (Yuan, 2017). However, these elite cultivars in our study did not show as high a seed-set percentage ratio as expected, even under normal conditions. Yang and Zhang (2009) argued that because of poor grain filling of later-flowering inferior spikelets (compared with the earlier-flowering superior spikelets), super hybrid rice fails to achieve its high yield potential. In our study, mean seed-set percentage in 2016 was 13.0% lower than that in 2015 at higher N levels but only 9.6% lower at lower N levels. These results suggest that higher N input may exacerbate a decrease in seed-set percentage of super hybrid rice cultivars under high temperature.

Prior to this study, few field experiments have attempted to elucidate the mechanism by which N management could alleviate the damaging effects of high temperature on rice grain yield. Previous studies conducted using controlled temperature chambers showed that high N fertilizer application could contribute to improvements in the number of panicles, spikelets per panicle, or grain weight in plants under high-temperature stress. Possible mechanisms could be a higher photosynthetic rate in flag leaves, greater root oxidation activity, or crucial enzymes involved in the sucrose-to-starch metabolic pathway in grains (Duan et al., 2013). However, in this study, no significant differences in grain weight or panicle numbers were observed (data not shown) across 2 years under different N treatments. The differences between studies may be attributable to the timing of the high temperatures. In our study, high temperature occurred during the flowering stage.

It should be noted that relatively higher numbers of spikelets per panicle were observed in those cultivars exposed to high temperature and higher N application levels than those at lower N levels (Figure 3A,B). The reason for this finding may be related to a higher number of primary and secondary branches. Liu et al. (2005) reported that primary and secondary branch differentiation was prolonged by higher N levels, and the number of branches increased accordingly, leading to an increase in maximum differentiated floret number per panicle. In the 2 years of our study, a larger amount of N was applied at panicle initiation in higher N treatments than in lower N treatments. Zhou et al. (2017) argued that postponing N application could reduce the degenerated number of secondary branches and increase the differentiation of spikelets, thus leading to an increase in the number of spikelets per panicle. In our study, spikelets per panicle was significantly related to N level and temperature (*P* < 0.01). Higher N levels may inhibit the number of degenerated spikelets under high temperature. Therefore, it seems that production of a higher number of spikelets per panicle is the key factor that contributes to reducing yield loss under high temperature by increasing N application levels.

## Author Contributions

YZ and KL initiated and designed the research, analyzed the data, and wrote the manuscript. KL, JD, and JL performed the experiments. XW, BL, and XT revised and edited the manuscript and also provided advice on the experiments.

## Conflict of Interest Statement

The authors declare that the research was conducted in the absence of any commercial or financial relationships that could be construed as a potential conflict of interest.
